# Supplementary material for: A Quantum Framework for Protein Binding‐Site Structure Prediction on Utility‐Level Quantum Processors
Source: Adv Sci (Weinh). 2025 Nov 28;13(12):e13641. doi: 10.1002/advs.202513641 (PMC12948255; doi:10.1002/advs.202513641)
Supplement: Supplementary file 1 — Supporting Information [file ADVS-13-e13641-s001.pdf]

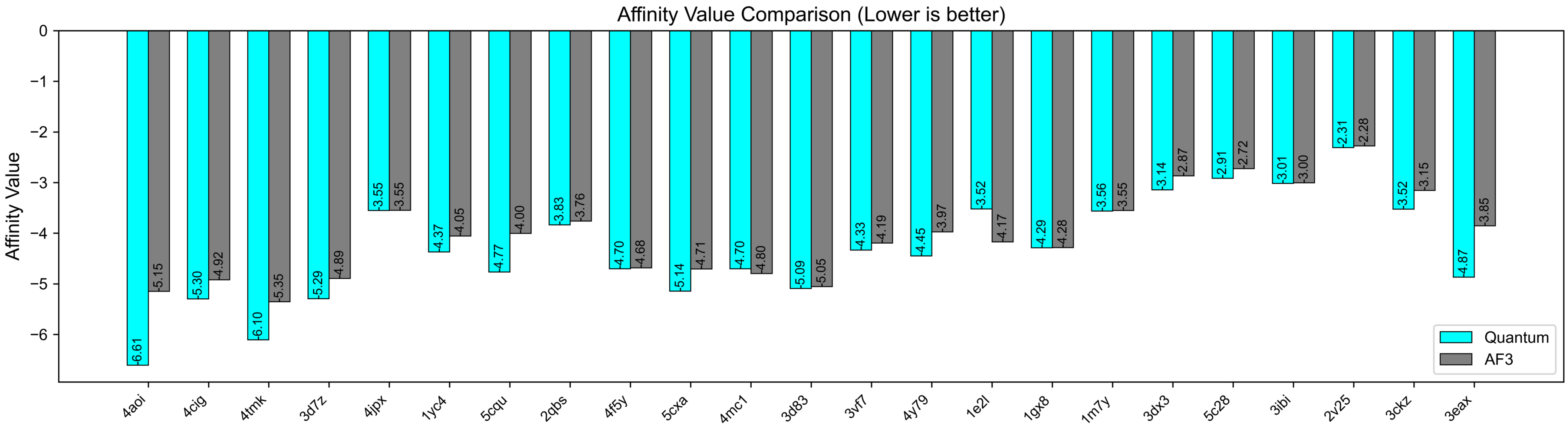

Figure S1: RMSD comparison between quantum and AF3 predictions. In 18 out of 23 test cases, quantum-predicted structures exhibit lower RMSD values relative to experimental structures compared to AF3, indicating improved geometric fidelity.

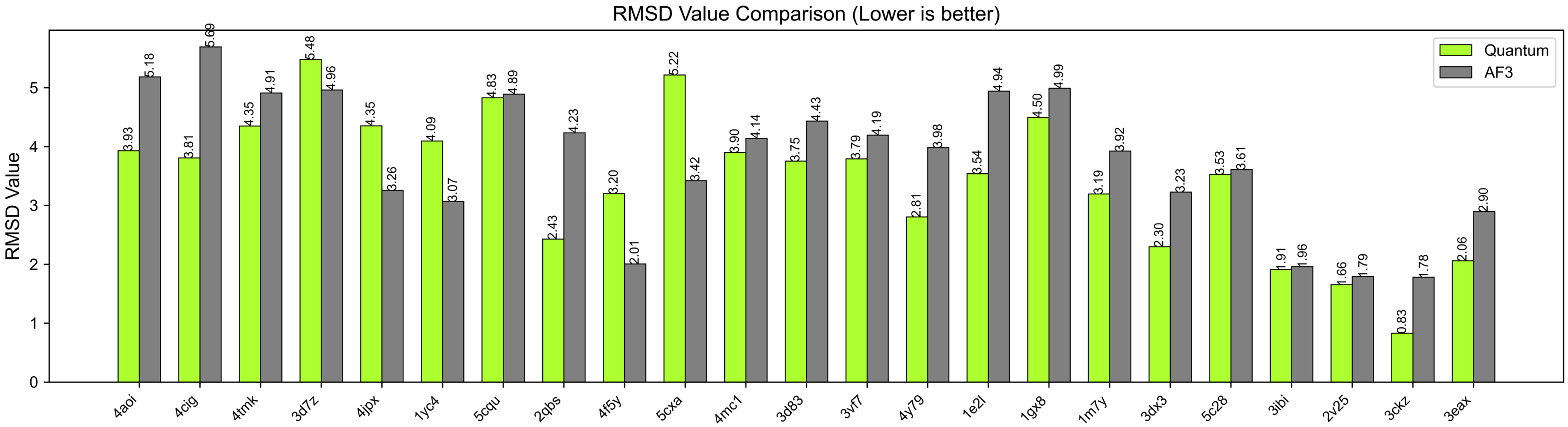

Figure S2: Docking affinity scores of quantum vs. AF3-predicted structures. Quantum structures achieved more favorable (lower) binding free energies in 21 of 23 cases, indicating better predicted protein-ligand interactions than AF3.

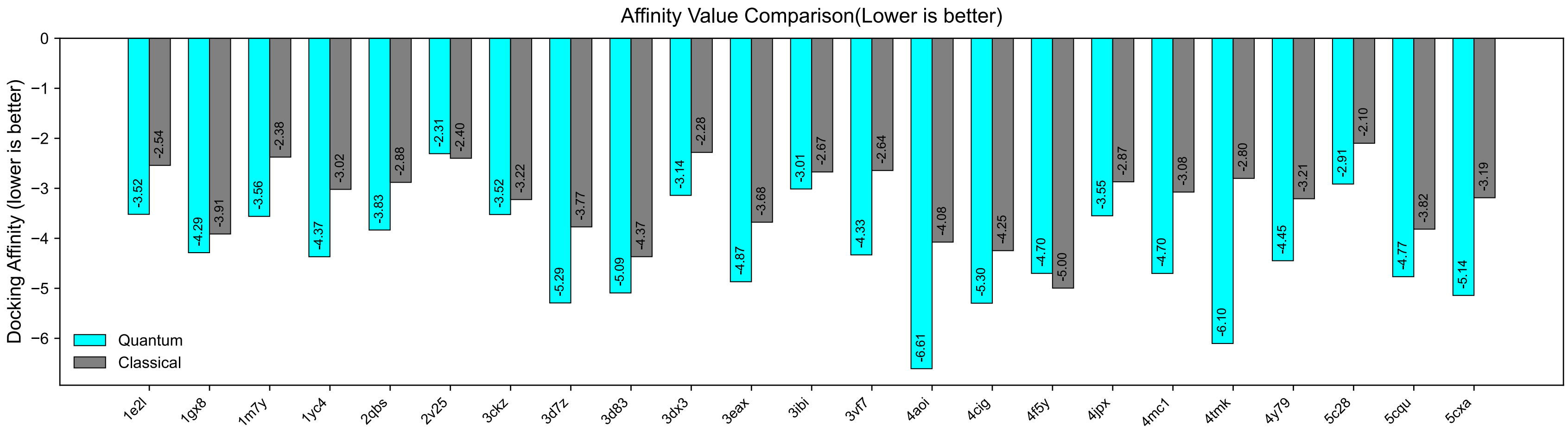

Figure S3: Docking affinity scores of quantum vs. classical-predicted structures. Quantum-predicted structures achieved more favorable (lower) binding free energies in 21 of 23 cases, indicating better predicted protein-ligand interactions and enhanced energetic realism compared to classical models.

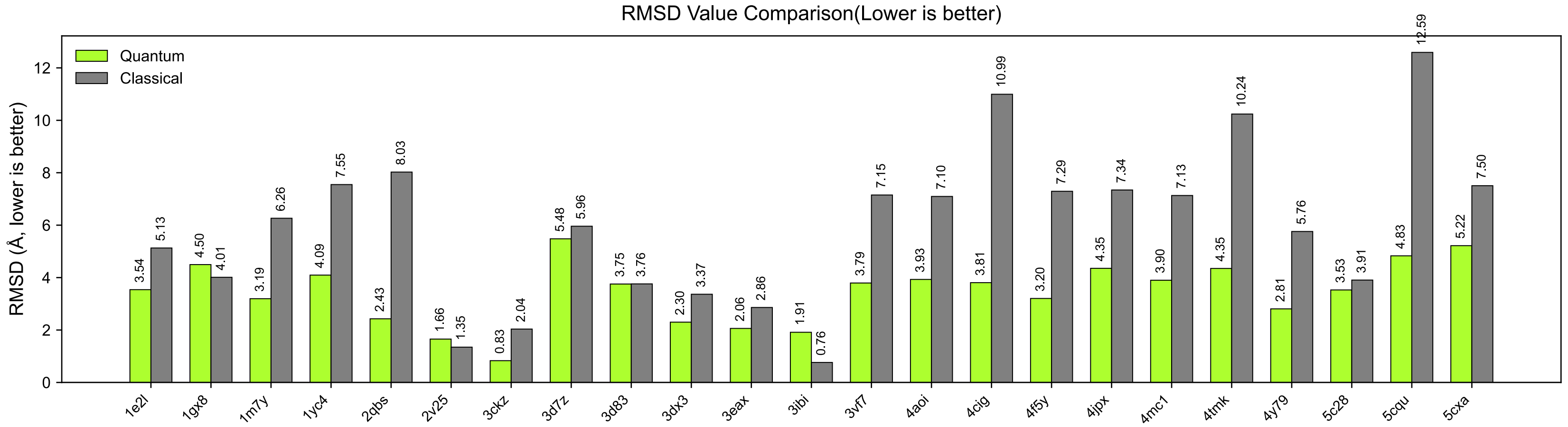

Figure S4: RMSD comparison between quantum and classical predictions. In 23 benchmark fragments, quantum-predicted structures exhibit lower RMSD values relative to experimental structures compared to classical models, indicating improved geometric fidelity and backbone stability, particularly in flexible or short-chain regions.

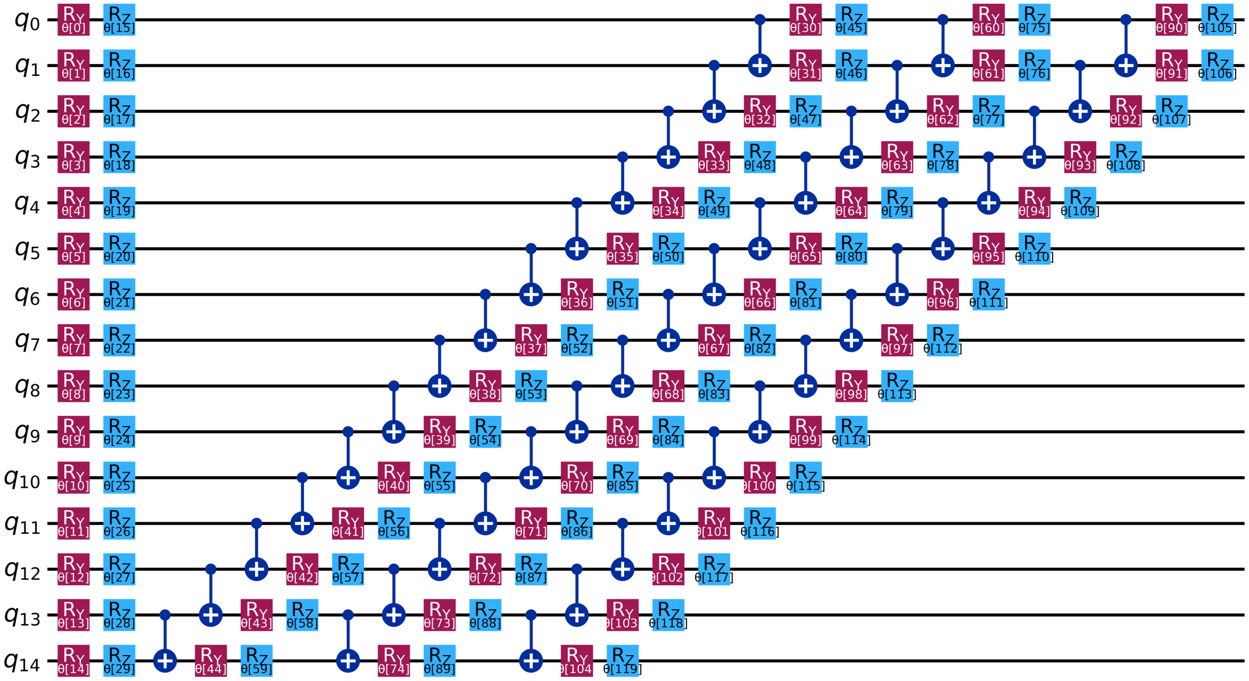

Figure S5: Parameterized quantum circuit design used in VQE. The ansatz follows the EfficientSU2 structure with alternating entanglement and rotation layers, enabling expressive quantum state preparation for structure optimization.

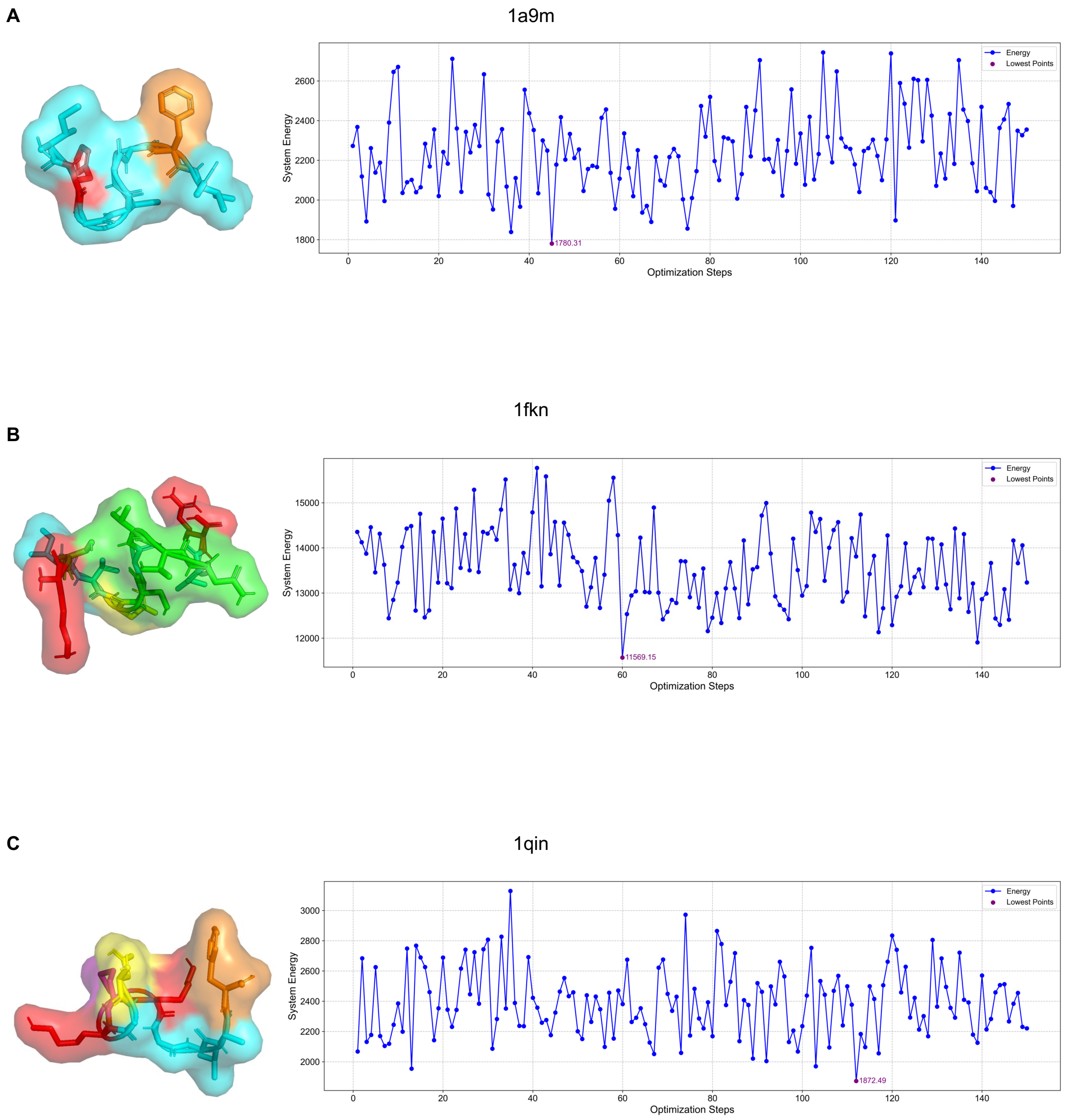

Figure S6: Predicted structures and energy profiles for 1a9m, 1fkn, and 1qin. Energy trajectories during VQE optimization are shown alongside the corresponding low-energy conformations for each fragment.

A

3ans

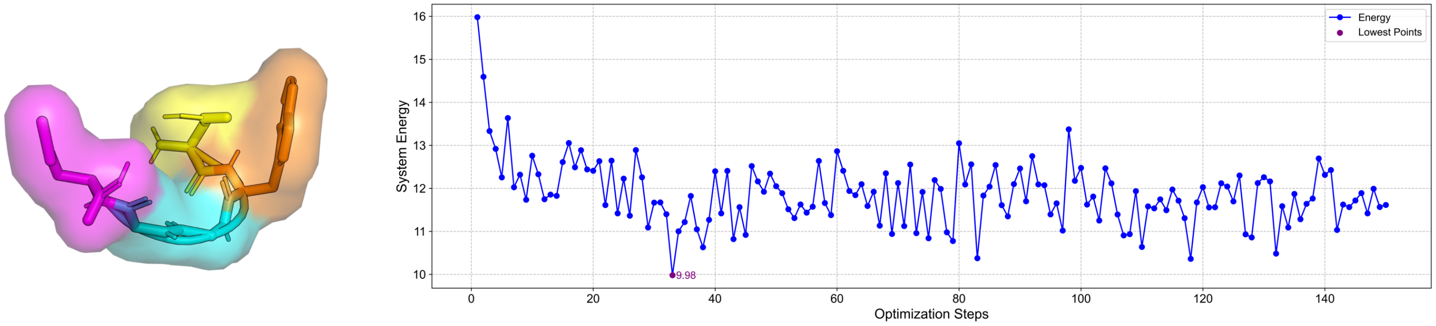

B

3b26

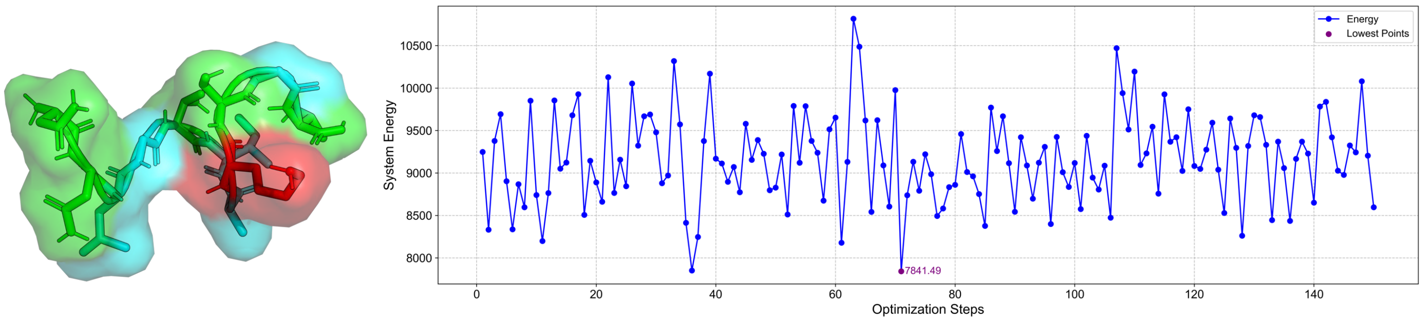

Figure S7: Predicted structures and energy profiles for 3ans and 3b26. Conformations sampled during VQE convergence illustrate how quantum-optimized structures correspond to energy minima.

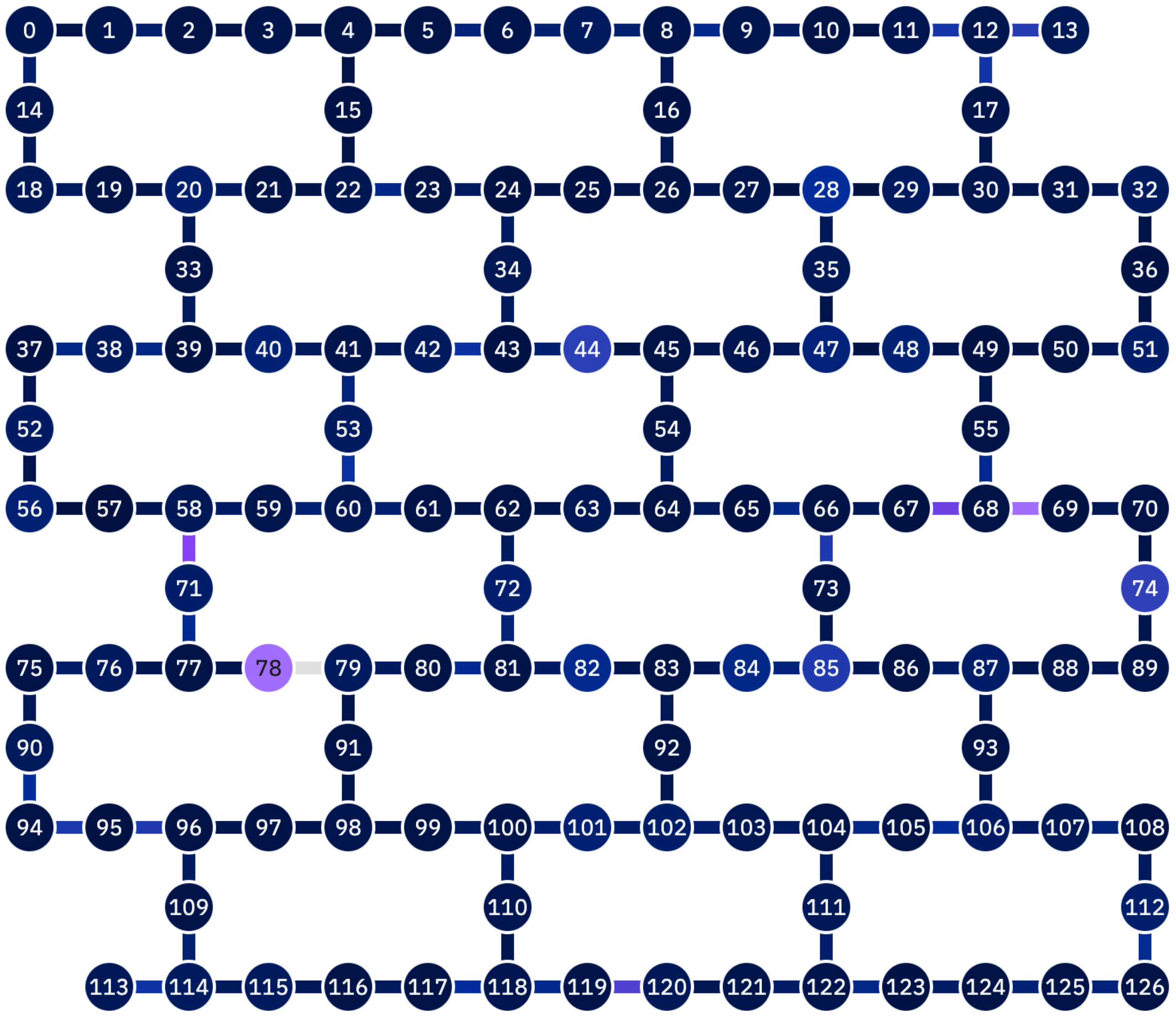

Figure S8: Coupling map of the IBM-Cleveland Clinic quantum processor. Conformations sampled during VQE convergence illustrate how quantum-optimized structures correspond to energy minima.

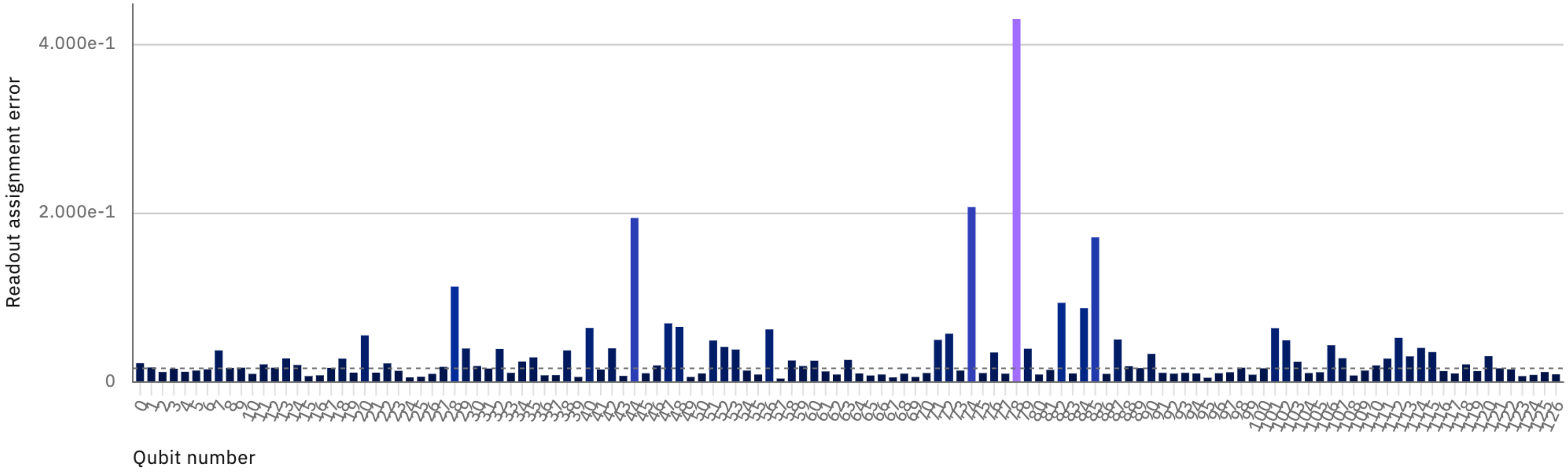

Figure S9: Raw noise statistics distribution of the IBM-Cleveland Clinic quantum processor.

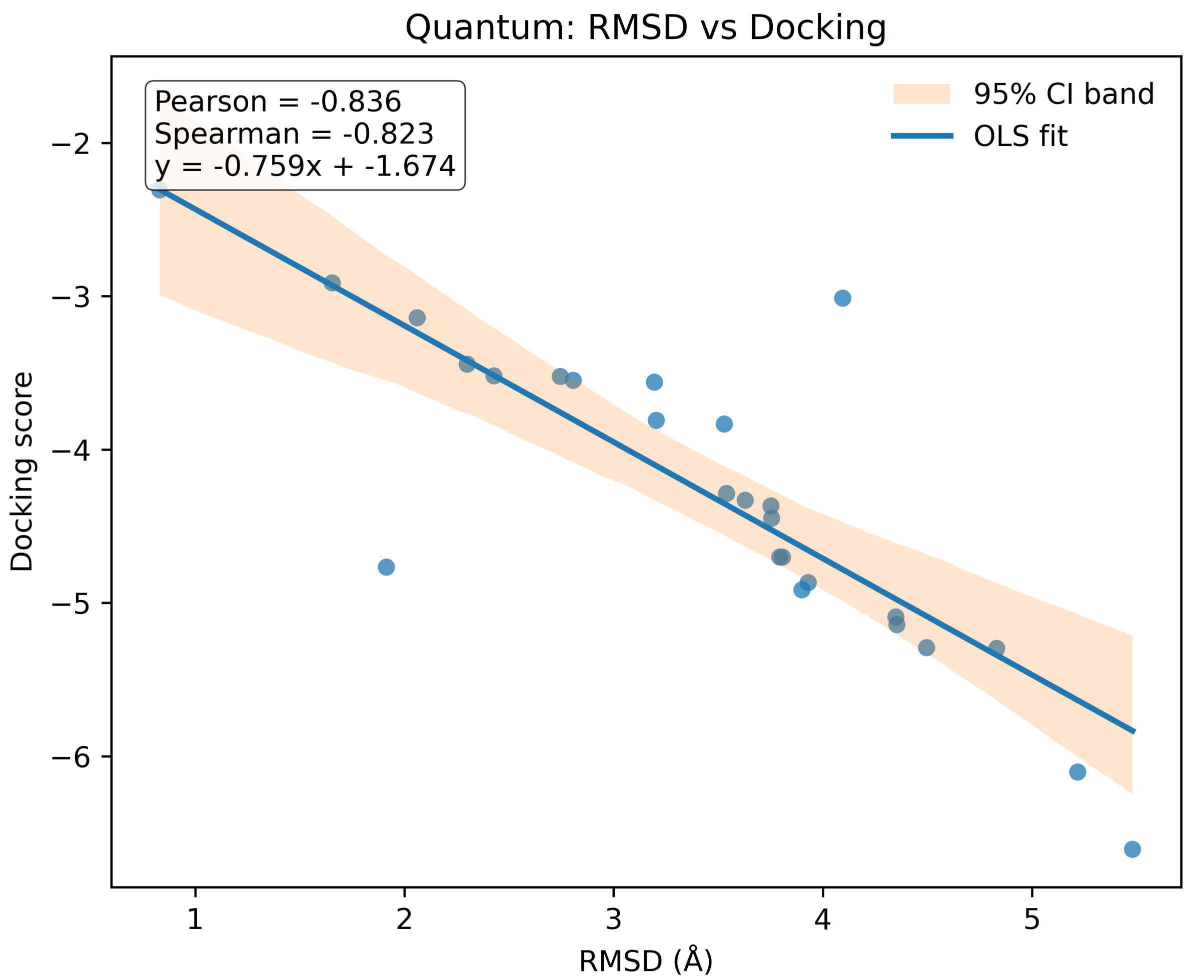

Figure S10: Correlation analysis between RMSD and docking score for the quantum-predicted structures. A strong negative correlation is observed, indicating that lower RMSD (i.e., higher structural accuracy) corresponds to stronger docking affinity. The correlation coefficients (Pearson = −0.836, Spearman = −0.823) confirm a statistically significant relationship between geometric and energetic consistency. Shaded areas represent the 95% confidence intervals.
